# Structural Transformations in the Thermal Dehydration of [Cu_2_(bpa)(btec)(H_2_O)_4_]_n_ Coordination Polymer

**DOI:** 10.3390/molecules24091840

**Published:** 2019-05-13

**Authors:** Laura Bravo-García, Edurne S. Larrea, Beñat Artetxe, Luis Lezama, Juan M. Gutiérrez-Zorrilla, María I. Arriortua

**Affiliations:** 1 Departamento de Mineralogía y Petrología, Facultad de Ciencia y Tecnología, Universidad del País Vasco, UPV/EHU, Sarriena s/n, 48940 Leioa, Spain; laura.bravo@ehu.eus (L.B.-G.); edurne.serrano@ehu.eus (E.S.L.); 2 Departamento de Química Inorgánica, Facultad de Ciencia y Tecnología, Universidad del País Vasco, UPV/EHU, Sarriena s/n, 48940 Leioa, Spain; benat.artetxe@ehu.eus (B.A.); luis.lezama@ehu.eus (L.L.); 3 BCMaterials, Basque Center for Materials, Applications and Nanostructures, Edf. Martina Casiano, UPV/EHU Science Park, 48940 Leioa, Spain

**Keywords:** crystal structure, topological study, 1,2-Bis(4-pyridyl)ethane, thermochromism

## Abstract

Reactions between pyridinic ligands such as 1,2-bis(4-pyridyl)ethane (*bpa*) and transition metal cations are a very widespread technique to produce extended coordination polymers such as Metal-Organic Frameworks. In combination with a second ligand these systems could present different topologies and behaviors. In this context, the use of 1,2,4,5-benzenetetracarboxylic acid (*H_4_btec*) gave us a novel 2D compound, [Cu_2_(bpa)(btec)(H_2_O)_4_]_n_ (**1**), which was prepared by microwave-assisted synthesis and structurally characterized by means of single crystal X-ray diffraction. Its thermal behavior was analyzed through thermogravimetric analysis and variable temperature powder X-ray diffraction, concluding that thermal stability is influenced by the coordination water molecules, allowing two sequential thermochromic phase transformations to take place. These transformations were monitored by electronic paramagnetic resonance spectroscopy and magnetic susceptibility measurements. In addition, the crystal structure of the anhydrous compound [Cu_2_(bpa)(btec)]_n_ (**1.ah**) was determined. Finally, a topological study was carried out for the *bpa* ligand considering all the structures deposited in the Cambridge Structural Databased. More than 1000 structures were analyzed and classified into 17 different topologies, according to the role of the ligand.

## 1. Introduction

Materials consisting of metal ions or clusters that are linked by polyfunctional organic ligands can lead to extended open frameworks with permanent porosity, well known as Metal Organic Frameworks (MOFs) [1,2]. Their structural features, including large cavities and large surface areas, have opened a wide range of applications [3,4,5,6] such as gas storage [7,8,9,10], gas separation [11,12,13], drug delivery [14,15,16], chemical sensing [17,18], heterogeneous catalysis [19,20,21] and biomedical imaging [22,23]. In this context, there is a general interest among the scientific community in making the synthesis of these materials cleaner and more effective. Thus, synthesis by non-conventional methods such as mechanochemical, solvent-free heating, microwave-assisted synthesis or sonochemistry [24] is an increasingly widespread strategy nowadays.

Among the different combinations of metal/organic ligand, the simultaneous use of aromatic polycarboxylates and *N*,*N*′-donor spacers seems to be an effective strategy to obtain these type of materials [25,26,27], because it increases the topological variability, increasing the possible applications. Furthermore, this broad range of topologies boosts the possibilities of a positive response to external stimuli [28]. Solid-state transformations triggered by a given external stimulus applied on a responsive material have long been identified as a focus of attention because: (i) they might follow a different pathway from that in solution due to the constrains imposed by the structural architecture, affording different products in comparison to those achievable by following alternative routes; and (ii) they allow for tuning the functionality of such responsive materials by controlling the applied stimulus. This feature makes them suitable candidates for a wide range of applications, including switches, memories, storage devices and sensors. 

Concerning coordination polymers, these materials can be classified into two groups according to their structural response. Robust frameworks do not present important structural changes as a result of the applied stimulus. Conversely, dynamic transformations are those in which the external stimulus (e.g., light, heat, mechanical force, removal or exchange of chemical guests) involves the rupture/formation of coordinative bonds, and occasionally, modifications in the oxidation state of the metal centers. These modifications usually imply the extensive rearrangement of the network of weak intermolecular interactions that stabilize the crystal packing, and hence they often result in remarkable changes of physical properties such as color, magnetism, luminescence, or porosity [29,30]. Among these phase transitions, those in which single crystal behavior is retained throughout the process (i.e., single-crystal-to-single-crystal transformations, SCSC) are the most popular, because they offer an incomparable tool for monitoring how the location of atoms varies within the crystal packing upon the application of the external stimulus, and for assessing how a given property of interest can be modified and tuned as a consequence [31,32,33,34,35].

Thermally triggered solid-state transformations represent the most frequent group. Usually the thermal stimulus promotes the removal of guest solvent molecules located in the pores of robust frameworks, leaving them accessible and operative for gas sorption/separation, catalysis or sensing. However, dynamic transformations promoted by the release of hydration and/or coordination water molecules have recently been reviewed [36]. Examples include (i) drastic changes in magnetic properties of the antiferromagnetic CoCl_2_(1,4-dioxane)(H_2_O)_2_, which exhibits ferromagnetic coupling in its anhydrous form [37]; and (ii) reversible rupture of the stable [Cu(5-bromonicotinate)_2_(H_2_O)_2_]_n_ paddle-wheel with associated solvatochromism [38]. 

In recent years, we have explored non-conventional synthetic methods for the preparation of supramolecular coordination networks that display interesting thermostructural behavior. Previous research indicated the correlation between the synthetic procedures (microwaved-assisted synthesis vs. sonochemistry) employed and the final dimensionality of the network in copper(II) coordination frameworks containing 1,3-benzenedicarboxylic acid and 1,2-bis(4-pyridyl)ethane (*bpa*) ligands [39]. Additional studies on the reversible thermal dehydration of the 1,2,4,5-benzenetetracarboxylic acid (*H_4_btec*) in the ionic [Cu(H_2_dpmd)_2_][H_2_btec] (H_2_dpmd = dipyridilmethanediol) framework afforded drastic structural changes with associated solvatochromism. This process implied a considerable decrease on the crystallinity of the sample that could be reverted with the addition of water. The nearly amorphous nature of the dehydrated phase avoided further structural elucidation [40]. To extend these studies, we combined the pyridinic *bpa* ligand of the former work with the thermoresponsive *H_4_btec.*

Herein, we report on the thermostructural behavior of the [Cu_2_(bpa)(btec)(H_2_O)_4_]_n_ (**1**) coordination polymer, which undergoes two sequential solvatochromic phase transformations upon gradual removal of coordination water molecules. The structure of the anhydrous [Cu_2_(bpa)(btec)]_n_ (**1.ah**) was determined by single-crystal X-ray diffraction and phase transformations analyzed by electron paramagnetic resonance spectroscopy and magnetic susceptibility measurements. The reversibility of such transitions has also been addressed. In addition, all the structures containing the *bpa* ligand that have been deposited in the Cambridge Structural Database have been analyzed and classified.

## 2. Results and Discussion

### 2.1. Synthesis and Preliminary Characterization

In a previous publication [39], some of us explored the effect of selecting different non-conventional synthetic methods for the reaction between *bpa* and benzenedicarboxylate (*m-bdc*). In the course of these studies, microwave-assisted synthesis was found to be the most efficient method for the prepration of frameworks with higher dimensionality. Therefore, the same synthetic method employed for [Cu_4_(m-bdc)_4_(bpa)_2_dmf]·dmf was used, but with *btec* as the polycarboxylic ligand instead. In this case, microwave-assisted synthesis provides a mixture of both blue crystals of the predominant phase **1** and a few green crystals of **1.ah**. The same result was obtained for different crystal batches. Crystals were separated manually under a polarized light microscope.

Compound **1** and **1.ah** were firstly identified by infrared spectroscopy (FT-IR). The FT-IR spectra, together with the list of the most intense bands and their assignations, are shown in Figure S1 and Table S1. Both spectra display the characteristic bands of both the *bpa* and *btec* ligands, as exemplified by the presence of the N=C stretching vibration band at 1300 cm^−1^ and a set of different overlapped signals around 1630 cm^−1^ related to asymmetrical and symmetrical stretching of C=O bonds from carboxylate groups, which could indicate the presence of *btec* ligands in different coordination modes (monodentate, chelating, bridging) [41]. However, the wide band centered at ca. 3082 cm^−1^, which corresponds to the stretching O–H vibrations from water molecules, is narrowed significantly when going from **1** to **1.ah**. Although the O–H band can still be observed in the spectrum of **1.ah** because of the humidity absorbed by the KBr matrix, it could suggest a lower water content in this compound in comparison to **1**.

### 2.2. Crystal Structures

Compound **1** crystallizes in the triclinic space group *P*-1 and its asymmetric unit contains one Cu(II) ion, one half of the *bpa* and one half of the *btec* ligand and two coordination water molecules (Figure S2). Both *bpa* and *btec* are located at an inversion center. The coordination environment of the metal center in a distorted octahedral geometry is CuNO_5_, with three oxygen atoms of carboxylate groups belonging to two different ligands, one in a monodentate mode and the other one in a chelating mode, one nitrogen atom from the pyridinic ligand and two coordination water molecules. Bond lengths and angles, together with the values for continuous shape measurements (CSM) [42] are summarized in Table 1. Each carboxylate ligand acts as a four-connected node between four metal centers forming metal-carboxylate chains running along the [100] direction, whereas the *bpa* ligand connects these chains to form covalent layers in the (01–2) plane. 

**Figure 1:**
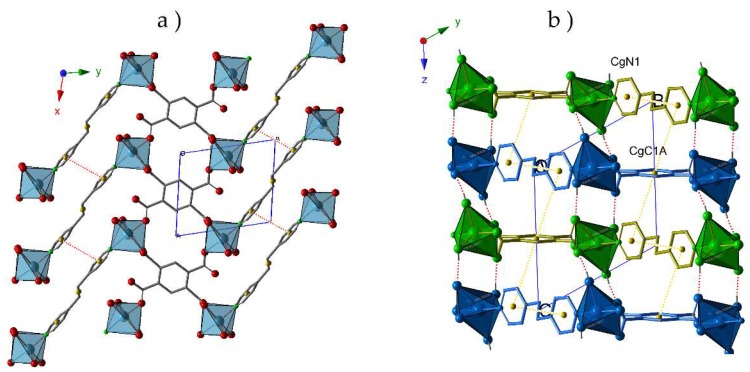
(**a**) View of the hybrid layers in **1** parallel to the (01–2) plane. The π-π interactions are depicted as red dotted lines; (**b**) View of the staking of the layers along the x axis. Hydrogen bonds are represented as red dotted lines, whereas the interactions between aromatic rings are depicted as yellow dotted lines. CgN1: N1, C2, C3, C4, C5, C6; CgC1A: C1A, C2A, C3A, C1A_d, C2A_d, C3A_d.

Thus, the crystal packing of compound **1** exhibits a bidimensional character (Figure 1a) where the π-π interactions between the aromatic rings of different pyridinic ligands (N1···N1) contribute to reinforce these layers. As shown in Figure 1b, the staking of these layers is antiparallel along the *z*-axes, and proceeds through i) T-type π-π interactions between C1A and N1 rings from *btec* and *bpa* ligands, respectively; and ii) hydrogen bonds established between the coordination water molecules and O atoms from carboxylate groups (O1W-H1WA···O1A, O1W-H1WB···O4A and O2W-H2WA···O2A). Distances and angles of these supramolecular interactions are summarized in Table 2. 

The anhydrous **1.ah** shows great differences with respect to the coordination environment of the metal centers in **1** and the arrangement of the ligands. Compound **1.ah** crystallizes in the triclinic space group *P*−1 and its asymmetric unit contains one Cu(II) ion, and half a ligand of each type (Figure S3). The loss of coordination water molecules promotes the formation of a centrosymmetric copper (II) dimer, where each metal center displays a CuNO_4_ environment with two bridging carboxylate ligands in a μ_2_-η^1^:η^1^ coordination mode, a third bridging carboxylate ligand in a μ_2_-η^2^ fashion and N atoms from *bpa* ligands completing the distorted pentacoordinated geometry. Bond lengths and angles together with the values for CSM are summarized in Table 3. 

**Figure 2:**
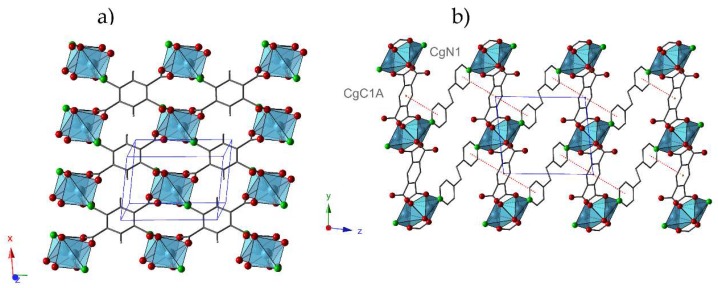
(**a**) View of the hybrid layers in **1.ah** parallel to the (001) plane; (**b**) View of the staking of the layers along the x axis. Interactions between aromatic rings are represented as dotted red lines. CgN1: N1, C2, C3, C4, C5, C6; CgC1A: C1A, C2A, C3A, C1A_c, C2A_c, C3A_c.

In addition, this structure results in an increase of the dimensionality of the framework in comparison with **1**, because Cu(II) dimers in **1.ah** can be visualized as being constituted by linking different metal centers located in adjacent layers in **1**. In **1.ah**, the *btec* ligand acts as a tetra-connector node forming layers parallel to the (001) plane (Figure 2a). These layers are stacked along the z-axis, where *bpa* ligands play the role of covalent bridge between dimers resulting in a three-dimensional structure (Figure 2b). The interactions between the aromatic rings of different types of ligands (N1···C1A) contribute to reinforce the network (Table 4).

### 2.3. Thermal Behavior

**Figure 3:**
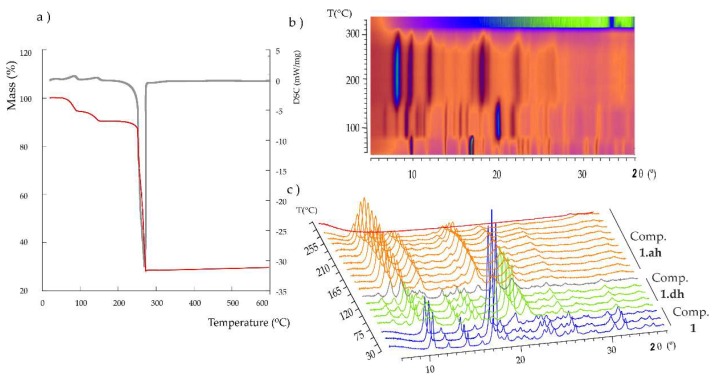
(**a**) TGA/DTA curves for **1** (exo down)**.** Variable temperature PXRD analysis for **1** from 30 °C to 315 °C (**b**) top 2D view and (**c**) 3D view.

The thermal stability of **1** was studied by thermogravimetric analyses (TGA/DTA) carried out under synthetic air atmosphere in the temperature range of 25–500 °C at a heating rate of 5 °C/min. As observed in Figure 3a, thermal decomposition proceeds via three different stages. The first mass loss below 100 °C corresponds to a dehydration process and it implies the release of one water molecule [%mass, calc. (found): 5.6 (5.3)]. The loss of the second coordination water molecule at ca. 160 °C [%mass, calc. (found): 5.6 (4.8)] results in the anhydrous phase, which is stable up to 220 °C. Above this temperature, an exothermic process takes place, which is associated with the combustion of the organic ligands and leads to the final residue at ca. 300 °C [% mass calc. (found) for CuO: 25.0 (28.0)]. The fact that the release of the two coordination water molecules takes place in two well-differentiated stages could be easily explained based on the crystal structure of **1**. The first step implies the release of the axial water molecule (OW2), because it exhibits a much longer Cu–O bond length (2.304 Å) in comparison with that shown by the second water molecule (Cu–OW1: 1.975 Å). Furthermore, the latter species establishes two O–H···O type hydrogen bonds, as well as an additional C–H···O type interaction with adjacent carboxylate groups, whereas the former only forms one O–H···O bond (Table 2).

Variable-temperature powder X-ray diffraction (termodiffractometry, TXRD) experiments were carried out from 30 to 500 °C at steps of 15 °C (Figure 3b) to determine the potential phase transformations in **1** upon thermal dehydration and the subsequent range of thermal stability. These results revealed a dynamic thermostructural behavior for **1**. The parent hydrated compound transforms into a second partially dehydrated phase (**1.dh**) upon release of the first coordination water molecule above 75 °C, as indicated by the clear modifications in either the positions and relative intensity of most of the diffraction maxima in the *2*θ = 5–30° angle range (Figure 3c). More specifically, instead of the most intense diffraction maxima at 10.6 and 18.0° for **1**, those at 10.0, 21.2 and 23.0° became predominant in the spectrum of the partially dehydrated phase. It is also worth noting the presence of two maxima of mid-to-low intensity at low *2*θ values (7.4 and 8.7°) for the latter phase. A totally anhydrous crystalline phase is completely formed at 150 °C and keeps stable up to 250 °C, above which the diffraction maxima lose intensity until full amorphization is reached at 285 °C. This temperature is in good agreement with the results obtained from the TGA analyses, which show that the thermally stable anhydrous phase starts decomposing at 250 °C. The intensity of the diffraction maximum located at 8.8° increases significantly when going from the partially dehydrated phase to the anhydrous form, whereas those at 10.4, 12.9, 19.3 and 23.6° became the most intense. The final residue at 330 °C was identified as monoclinic *C2/c* CuO, also known as tenorite [43].

Taking into account the structural similarities between **1** and **1.ah**, we hypothesized whether the latter compound could be prepared by thermal dehydration of the former. Thus, a crystalline sample of **1** was heated in the oven at 200 °C for 1 h, and immediately afterwards it was analyzed by powder XRD. There was a good agreement in the profile fitting procedure carried out between the experimental spectrum and the cell unit parameters obtained in the single crystal XRD studies (Figure S4), revealing that **1.ah** can be directly obtained by total thermal dehydration of **1**. Cell parameters, the displacement of the sample, the shape of the diffraction maxima, as well as the angular evolution of the half-height width were refined using FULLPROF [44]. Agreement factors of the fitting are summarized in Table S2. Similarly, we tried to elucidate the crystal structure of the partially dehydrated phase by heating single crystals of **1** at 90 °C for 1 h. Unfortunately, despite our efforts, crystals did not preserve their integrity, and we were not able to perform a full single-crystal XRD data acquisition. Figure S5 shows the experimental powder X-Ray diffraction patterns in comparison to those simulated from single crystal data for both compounds **1** and **1.ah**. The good agreement between experimental and simulated patterns indicate the presence of pure crystalline phases in bulk samples. 

### 2.4. Reversibility of Thermally Triggered Phase Transformation and EPR Espectroscopy

**Figure 4:**
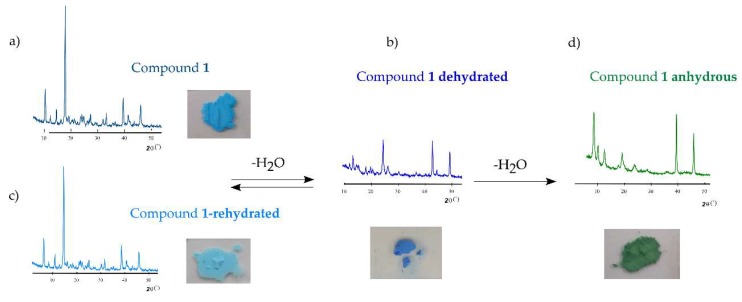
Powder X-ray diffraction patterns and photographs displaying the color of powdered samples of **1** (**a**) at room temperature, (**b**) after being heated at 90 °C, (**c**) after being heated at 90 °C and rehydrated in open air conditions, (**d**) after being heated at 200 °C. The reversibility of such transformations is also indicated.

The reversibility of thermally triggered structural transformations that **1** undergoes and the associated solvatochromism (thermochromism) was studied by XRD (Figure 4). A powdered light blue sample of **1** was heated at 90 °C in an oven and a clear color change to dark blue was observed as a result of modifications in the coordination environment of the Cu(II) center promoted by the thermal evacuation of the first coordination water molecule. When the partially dehydrated phase is exposed to ambient moisture, it reverts to the parent phase **1** upon re-hydration, as evidenced by its color change and the registered powder XRD pattern. As mentioned above, a second sample of **1** was heated to 200 °C, and the anhydrous **1.ah** was obtained. The color change from blue to green arises not only from the removal of coordination water molecules, but also from modifications in the coordination modes of *btec* ligands. In this case, the dehydration is not reversible because no rehydration is observed neither when the solid is exposed to ambient moisture, nor when it is soaked in water. 

**Figure 5:**
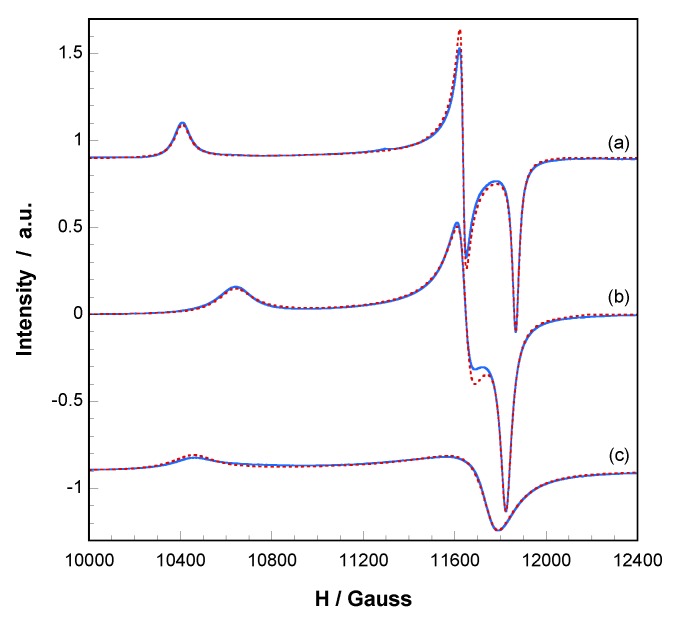
Q-band EPR spectra recorded at room temperature on polycrystalline samples of (**a**) compound **1**, (**b**) heated at 90 °C and (**c**) heated at 200 °C.

These phase transformations and their reversibility were monitored by electron paramagnetc resonance spectroscopy (EPR). X and Q band EPR measurements were carried out at several temperatures in the 4.2–298 K range. The X-band EPR spectra of **1** and the corresponding dehydrated phases exhibit nearly axial symmetries for the g tensor in the ΔMs = ± 1 region, but an appreciable extent of rhombicity can be detected operating at Q-band (Figure 5). The spin Hamiltonian parameters were estimated by comparison of the experimental spectra with those obtained by a computer simulation program working at the second order of the perturbation theory. The best-fit results are represented as dashed lines in Figure 5.

The main components of the g tensor for **1** are g_1_ = 2.338, g_2_ = 2.091 and g_3_ = 2.051 (g_II_ = 2.338, g_⊥_ = 2.071, <g> = 2.160). These values are typical of Cu(II) ions in distorted octahedral environment in good agreement with the structural characteristics of the CuNO_5_ chromophore. In addition, the lowest g value deviates appreciably from the free electron value (g_e_ = 2.0023), indicating a d_x_2-_y_2 ground state which corresponds to that expected for an axially elongated octahedral Cu(II) ion [45].

After heating a sample of **1** at 90 °C, the spectrum continues displaying a single and well-defined signal, with rhombic g values of g_1_ = 2.287, g_2_ = 2.090 and g_3_ = 2.058 (g_II_ = 2.287, g_⊥_ = 2.074, <g> = 2.145). The reduction of g_II_ is in good agreement with the loss of the water molecule in apical position. As the axial field is removed, the copper ion attracts the equatorial ligands more strongly, the d_x_2-_y_2 orbital becomes more antibonding and the g_II_ value decreases [46]. It must be taken into account that, for systems with similar covalence degree, the g values depend mainly on the energy of the d-d transitions, following these Equations:

(1) $$g_{\mathrm{II}}{=g}_{e}-\frac{{8K}_{\mathrm{II}}^{2}\lambda_{0}}{\Delta_{1}}$$

(2) $$g_{⊥}{=g}_{e}-\frac{{2K}_{⊥}^{2}\lambda_{0}}{\Delta_{2}}$$

where λ_0_ is the free ion spin-orbit coupling constant (λ_0_ = −830 cm^−1^ for Cu^2+^), K_II_ and K_⊥_ are the covalence factors, and Δ_1_ and Δ_2_ are the energies of the d_xy_→d_x_^2^-_y_^2^ and d_xz,yz_→d_x_^2^-_y_^2^ transitions, respectively [47]. Diffuse reflectance UV-Vis spectra (Figure S6) showed that the center of gravity of the d-d transitions shift to higher energies when the first water molecule is removed. This effect can be visually observed in its color change (from a lighter to a darker blue) and it is in good agreement with the decrease of the <g> value. 

When the sample is heated at 200 °C, **1.ah** is obtained, and thus, a new EPR signal is registered showing different g values: g_1_ = 2.327, g_2_ = 2.075 and g_3_ = 2.069 (g_II_ = 2.327, g_⊥_ = 2.072, <g> = 2.157). The relative increase of the g_II_ component implies that the loss of the second water molecule is accompanied by changes in the coordination of the Cu(II) ions, which results in a slight increase of the equatorial distances [46]. This is in good agreement with the crystallographic data. In addition, it is worth noting that the Cu(II) hyperfine lines could not be resolved in any of these compounds and therefore extensive magnetic exchange is present in all of them. However, the G parameters, which are in the range 3.8–4.8, indicate that the local tetragonal axes of the molecules are aligned parallel or only slightly misaligned [45]. Therefore, it can be assumed that the calculated g values correspond to the molecular tensors and adequately reflect the characteristics of the environment of the Cu^2+^ ions in each compound. Moreover, the EPR spectra of compound **1** and the dehydrated phase at 90 °C remain practically unchanged over the temperature range 4.2–298 K, so the magnetic interactions should be of small magnitude in both compounds. On the contrary, the intensity of the EPR signal of compound **1.ah** decrease below 90 K, indicating that moderate antiferromagnetic exchange is operative between the copper(II) ions of the dimeric entity. Thus, temperature dependent magnetic measurements were carried out.

**Figure 6:**
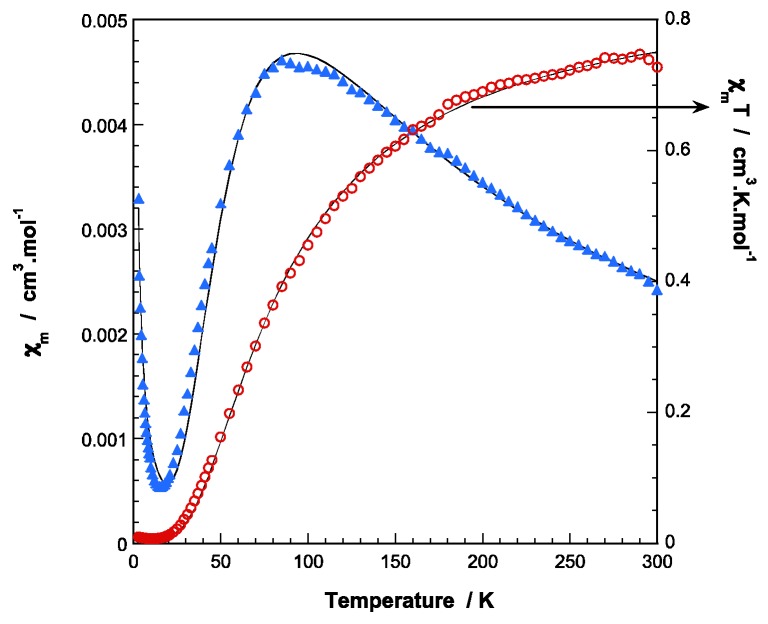
Thermal evolution of *χ_m_* (blue triangles) and *χ_m_T* product (red circles) for **1.ah**. The solid line corresponds to the best theoretical fit (see text for the details).

The magnetic susceptibility (*χ_m_*) of **1** reveals a Curie-Weiss behavior for the whole temperature range analyzed and the fit of the data to the corresponding expression leads to *C_m_* and θ values of 0.44 cm^3^K/mol and −0.2 K, respectively (Figure S7). Conversely, temperature-dependent susceptibility data registered for **1.ah** displays a maximum at ca. 90 K and decreases to 15 K upon cooling. Below this temperature, there is a drastic increase in magnetic susceptibility as the temperature decreases. The room temperature magnetic moment (*χ_m_T* = 0.73 cm^3^K/mol, *μ_eff_* = 3.42 BM) is considerably lower than that expected for two magnetically isolated copper (II) ions with g=2.16 (*χ_m_T* = 0.87 cm^3^K/mol, *μ_eff_* = 3.74 MB). The *χ_m_T* curve continuously decreases when cooling, until it almost vanishes at very low temperatures. These features are consistent with moderately strong antiferromagnetic exchange between the Cu(II) ions within the dimer. The behavior below 15 K could be explained by the presence of a small amount of monomeric Cu(II) species. According to these observations, the experimental data can be well fitted to the Bleaney and Bowers equation for a dinuclear Cu(II) complex [48] modified with an additional term to take into account the presence of non-coupled Cu(II) impurities following a simple Curie law with the same g factor:

(3) $$\chi_{m}=\frac{\left(1-\rho \right)Ng^{2}\beta^{2}}{kT(3+3\exp\left(-J/kT\right)}+\frac{\rho Ng^{2}\beta^{2}}{4kT}$$

where the singlet-triplet energy gap (2*J)* is defined by the Hamiltonian *H* = −2*J·S_1_·S_2_* (*S_1_* = *S*_2_ = 1/2), *ρ* is the percent of non-coupled component and other symbols have their usual meanings. A good fit to the data (solid lines in Figure 6) was obtained when g = 2.16, *J* = −52.4 cm^−1^, and *ρ* = 0.013, with an error R =2.4 × 10^−4^. According to the topology of the Cu···Cu dimeric entities in **1.ah**, different exchange pathways must be taken into account: i) two axial-equatorial monoatomic carboxylate oxygen bridges, ii) two carboxylate bridges in *syn-syn* mode. It is well known that axial–equatorial bridging modes between Cu^2+^ ions provide weak spin communication between magnetic d*_x_*_2−*y*2_ orbitals, thus the observed *J* value can be mainly assigned to the *syn-syn* bridges [49]. 

### 2.5. Topological Study of the bpa Ligand

**Figure 7:**
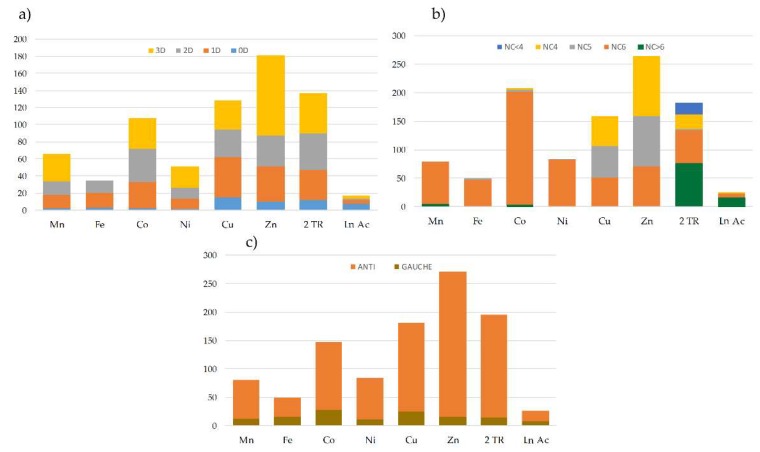
(**a**) Graphical representation of the number of structures with different dimensionalities (0D, 1D, 2D and 3D) containing *bpa* as a bridge between the different metal centers; (**b**) Graphical representation of the number of structures with different coordination environments (NC < 4, NC4, NC5, NC6, NC > 6) containing *bpa* as a bridge between the different metal centers; (**c**) Graphical representation of the number of structures with different conformational states (*anti* and *gauche*) containing *bpa* as a bridge between the different metal centers.

Although it exhibits limited coordination modes (monodentate or ambidentate), the *bpa* ligand usually acts as linker of metal centers leading to extended structures with very different topologies. An exhaustive bibliographic search in the Cambridge Crystallographic Database (CSD) [50] of structures containing transition metal ions, lanthanides and actinides provided us with a set of 1197 structures in which the *bpa* ligand plays a bridging role between metal centers. Figure 7 shows the general distribution of these structures according to their dimensionality (Figure 7a), the coordination number of the metal centers (Figure 7b), and the conformation of the ligand (Figure 7c). It must be highlighted that most of the structures found in the database are combinations of the *bpa* ligand with first raw transition metals in a hexacoordinated environment, and only a few examples belong to the families of 4d and 4f/5f metals. In all cases, the *anti* conformation of the *bpa* is clearly predominant over the *gauche* form (for representations of the extreme conformations see Figure S8).

**Figure 8:**
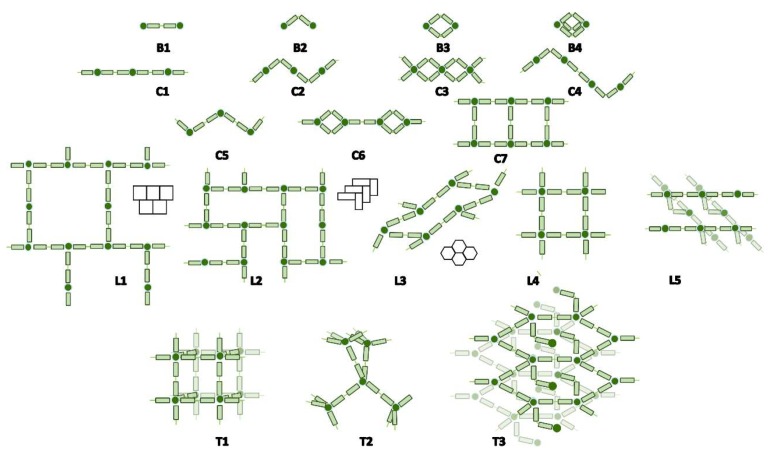
Schematic representation of the different topologies found in the CSD database for all the coordination compounds based on the *bpa* ligand.

Close inspection of the crystal structures showed four different ways in which the *bpa* ligand could contribute to the final dimensionality of the network. Beginning from the most frequent forms, these are: (i) zero connector or bridge (B); (ii) mono-dimensional connector or linear chain (C); (iii) bi-dimensional connector or layers (L); and (iv) three-dimensional network (T). These modes were classified according to their topology as displayed in Figure 8. Additionally, Table 5 compiles the number of structures of each possible topology, together with a brief description and a representative example for each case. The *bpa* ligand in both compounds **1** and **1.ah** reported in this work acts as a B1 linker, which increases the dimensionality of Cu(II)-btec systems from 1D to 2D for **1** and from 2D to 3D for **1.ah**.

## 3. Experimental

### 3.1. Materials and Methods

All solvents and chemicals were used as received from reliable commercial sources without further purification. The reactants 1,2-bis(4-pyridyl)ethane (*bpa*), 1,2,4,5-benzenetetracarboxylc acid (*H_4_btec*) and copper(II) nitrate 2.5 hydrate 99%, and the solvent *N*,*N*-dimethylformamide (DMF) 99.8% were purchased from Sigma-Aldrich, (Madrid, Spain) whereas nitric acid 65% (HNO_3_) was purchased from Panreac (Barcelona, Spain).

The FT-IR spectra were collected as KBr pellets (1%w of sample) on a Shimadzu FTIR-8400S spectrophotometer (Shimadzu, Kyoto, Japan) at room temperature in the 400–4000 cm^−1^ range. The thermogravimetric analysis (TG/DTA) was performed from room temperature to 600 °C at a rate of 5 °C min^−1^ on a SDT 2960 DSC-TGA NETZSCH STA 449F3 instrument (Netzsch-Geräte GmbH, Barcelona, Spain). The carbon, hydrogen and nitrogen contents were determined on a Euro EA 3000 CHN Elemental Analyzer (EuroVector, Milan, Italy). Thermodiffractometric (TDX) analysis was carried out in a Bruker D8 Advanced Theta-Theta diffractometer (Bruker, Madison, WI, USA) equipped with CuKα radiation (λ = 1.5418 Å) and HTK2000 Chamber with a Pt sample holder. The patterns were recorded from room temperature to 500 °C at steps of 15 °C every 10 min. Diffuse Reflectance studies were performed on a UV-Vis-NIR Varian Cary 500 spectrophotometer (Varian, Palo Alto, CA, USA). X-band EPR measurements were registered on a Bruker ELEXSYS 500 spectrometer equipped with a super-high-Q resonator ER-4123-SHQ (Bruker, Karlsruhe, Germany) and standard Oxford low-temperature devices. For Q-band studies, EPR spectra were recorded on a Bruker EMX system equipped with an ER-510-QT resonator (Bruker, Karlsruhe, Germany). The magnetic field was calibrated by a NMR probe and the frequency inside the cavity was determined with a Hewlett Packard 5352B microwave frequency counter (Palo Alto, CA, USA). Computer simulation: WINEPR-Simfonia, version 1.5, Bruker Analytische Messtechnik GmbH (Bruker, Karlsruhe, Germany). Temperature-dependent magnetic measurements were performed between 3 and 300 K with an applied field of 0.1T using a commercial MPMS3 SQUID magnetometer (Quantum Design, San Diego, CA, USA). The experimental susceptibilities were corrected for the diamagnetism of the constituent atoms by using Pascal tables [70].

### 3.2. Synthesis of [Cu_2_(bpa)(btec)(H_2_O)_4_]n (1) and [Cu_2_(bpa)(btec)]n (1.ah)

Solid 1,2,4,5-benzenetetracarboxylc acid (0.25 mmol) dissolved in DMF (10 mL) was added to a blue aqueous solution (10 mL) of Cu(NO_3_)_2_·2.5H_2_O (0.40 mmol) and 1,2-bis(4-pyridyl)ethane (0.20 mmol). The resulting mixture was transferred to a 100 mL CEM MARS5 XP-1500 plus microwave vessel and heated at 140 °C (800 W) for 1 h under autogenous pressure. After cooling down to room temperature, a mixture of blue prismatic crystals of [Cu_2_(bpa)(btec)(H_2_O)_4_]_n_ (**1**) and a few green prismatic crystals of [Cu_2_(bpa)(btec)_4_]_n_ (**1.ah**) were obtained. Crystals were separated manually under optical microscope. Yield for **1**: 100 mg, 79.25% based on Cu. Elemental analysis calc. for C_22_H_22_Cu_2_N_2_O_12_ (**1**): C, 41.06; H, 5.01; N, 4.35. Found: C, 41.60; H, 4.53; N, 4.94. FT-IR (cm^−1^): 3430 (m), 3082(m), 1620(s), 1600(s) 1580(s), 1490(m), 1410 (m), 1360(s), 890(m), 800(m), 770(m), 650(m). Compound **1.ah** can be obtained as a pure phase by heating **1** in an oven at 200 °C for 1h. Elemental analysis calc. for C_11_H_7_CuNO_4_ (**1.ah**): C, 47.06; H, 2.51; N, 4.99. Found: C, 46.90; H, 2.22; N, 5.11. FT-IR (cm^−1^): 3371(m), 1625(s), 1605(s), 1584(m), 1505 (w), 1462(m), 1359(s), 857(m), 836(m), 754(m), 672(m).

### 3.3. Single-Crystal X-Ray Diffraction

Single crystals of **1** and **1.ah** with dimensions given in Table 6 were selected under polarizing microscope and mounted on a MicroMounts^TM^ (MiTeGen, LLC, Ithaca, NY, USA). Intensity data were collected at 150 K on an Agilent Technologies Supernova single source diffractometer equipped with Cu-Kα (1.54184 Å) radiation and Atlas CCD detector (Agilent, Santa Clara, California, USA). Data frames were processed (unit cell determination, multi-scan absorption correction, intensity data integration and correction for Lorentz and polarization effects) using the CrysAlis Pro software package [71]. The structures were solved using OLEX2 [72] and refined by full–matrix least–squares based on F^2^ with SHELXL–2014/6 [73] as integrated in WinGX [74]. Thermal vibrations were treated anisotropically for heavy atoms. Hydrogen atoms of the organic ligands were placed in calculated positions and refined using a riding model with standard SHELXL parameters, except for those belonging to water molecules, which were located in the Fourier maps and O–H bond lengths manually restrained to 0.84(2)Å (DFIX). Details of crystal data and some features of the structural refinements are reported in Table 6. CCDC 1910260 (**1**) and 1910261 (**1.ah**) contains the supplementary crystallographic data for this paper. These data can be obtained free of charge from The Cambridge Crystallographic Data Centre via www.ccdc.cam.ac.uk/data_request/cif.

## 4. Conclusions

The thermally triggered phase transition of the coordination polymer [Cu_2_(bpa)(btec)(H_2_O)_4_]_n_ (**1**) (*bpa* = 1,2-bis(4-pyridyl)ethane, *btec* = 1,2,4,5-benzenetetracarboxylate) was analyzed in this work. The title compound, which involves the combination of a pyridinic and a polycarboxylic ligand, was prepared by microwave-assisted synthesis and presents a bidimensional covalent structure formed by isolated Cu(II) centers linked through organic ligands, as demonstrated by single-crystal X-ray diffraction studies. Variable-temperature powder X-ray diffraction revealed that **1** undergoes two sequential phase transformations upon gradual removal of coordination water molecules. These transformations involve thermochromism (from blue to green) as a result of modifications in the coordination sphere of Cu(II) centers. The loss of the first water molecule was found to be reversible, whereas the anhydrous form cannot revert to any of its parent phases by rehydration. Full dehydration leads to the anhydrous structure [Cu_2_(bpa)(btec)]_n_ (**1.ah**), which displays copper(II) dimers produced by changes in the coordination modes of the *btec* ligands and increases the dimensionality, from 2D to 3D, in comparison with the parent hydrated **1**. Variable temperature electronic paramagnetic resonance spectra are in good agreement with these transformations and magnetic susceptibility measurements conclude that there is a moderately strong antiferromagnetic exchange between the two Cu(II) ions within the dimeric entities in **1.ah**. Finally, making use of the Cambridge Structural Database, all the structures containing the *bpa* ligand (>1000 entries) were analyzed and classified into 17 different topologies according to the role of the ligand.

## Figures and Tables

**Table 1:** Bond lengths and angles for Cu(II) centers in **1**, together with the values for CSM.

Bond Lengths (Å)		Bond Angles (°)				
Cu1-N1	2.003(4)	N1-Cu1-O2A^ii^	89.4(2)		O1A^ii^-Cu1-O1W	108.7(1)
Cu1-O3A	1.970(3)	N1-Cu1-O1W	90.6(2)		O1A^ii^-Cu1-N1	91.6(1)
Cu1-O1W	1.975(4)	O3A-Cu1-O2A^ii^	86.0(2)		O2W-Cu1-N1	98.5(2)
Cu1-O2A^i^	2.023(3)	O3A-Cu1-O1W	93.2(2)		O2W-Cu1-O1A^ii^	155.3(1)
Cu1-O2W	2.304(4)	O1A^ii^-Cu1-O2A^ii^	55.9(2)		O2W-Cu1-O3A	84.4(1)
Cu1-O1A	2.508(4)	O1A^ii^-Cu1-O3A	84.2(2)		O2W-Cu1-O1W	93.7(1)
CSM Distortion	OC-6			TPR-6		
Cu1	3.814			14.855		

Symmetry codes: (i) x − 1, y, z; (ii) −1 + x, y, z. Ideal reference shapes: OC-6 = octahedron and TPR-6 = trigonal prism.

**Table 2:** Distances (Å) and angles (°) of supramolecular hydrogen bonding and π-π interactions in **1**.

**D-H···A**	**D···A**	**<D-H···A**	**D-H···A**	**D···A**	**<D-H···A**
O1W-H1WA···O1A^i^	2.655(5)	170(5)	C2 -H2···O2A^ii^	3.318(7)	174
O1W-H1WB···O4A	2.590(6)	156(6)	C6 -H6···O1A^i^	3.266(7)	145
O2W-H2WA···O2A^ii^	2.755(5)	172(6)	C7 -H7B···O1W^iii^	3.298(7)	132
**Cgi-Cgj**	**DC**	**ANG**	**DZ**	**DZ’**	**DXY**
CgN1-CgN1^iii^	3.843(3)	0.0(3)	3.605(2)	3.605(2)	1.331
CgC1A-CgN1^iv^	5.308(3)	88.3(3)	0.357(2)	4.914(2)	

Symmetry codes: (i) 1 − x, 1 − y,1 − z; (ii) 1 − x,1 − y,2 − z (iii) −x,2 − y,2 − z, (iv) 1 + x, −1 + y, z.

**Table 3:** Bond lengths and angles for Cu(II) centers in **1.ah**, together with the values for CSM.

Bond Length (Å)		Bond Angles (°)					
Cu1—O1A	1.930 (6)	N1-Cu1-O4A	92.7 (3)		O1A^i^-Cu1-N1		96.4 (3)
Cu1—O3A^ii^	1.970 (6)	O4A-Cu1-O1A	88.2 (3)		O1A^i^-Cu1-O1A		100.3 (3)
Cu1—O4A^iii^	1.985 (6)	O1A-Cu1-O3A	88.9 (3)		O1A^i^-Cu1-O3A		82.7 (3)
Cu1—N1	1.978 (8)	O3A-Cu1-N1	95.1 (3)		O1A^i^-Cu1-O4A		80.2 (3)
Cu1—O1A^i^	2.442 (9)						
**CSM distortion**	**vOC-5**			**TBPY-5**		**SPY-5**	
Cu1	2.584			2.405		2.755	

Symmetry codes: (i) –x + 1, −y + 1, −z; (ii) −x, −y + 1, −z; (iii) x + 1, y, z. Ideal reference shapes: vOC-5 = vacant octahedron; TBPY-5 = trigonal bipyramid; SPY-5 = square pyramid.

**Table 4:** Distances (Å) and angles (°) of supramolecular hydrogen bonding and π-π interactions in **1.ah**.

**D-H···A**	**D···A**	**<D-H···A**	**D-H···A**	**D···A**	**<D-H···A**
C2-H2···O4A^i^	2.979(11)	111	C6-H6···O3A^ii^	2.998(12)	118
**Cgi-Cgj**	**DC**	**ANG**	**DZ**	**DZ’**	**DXY**
CgN1-CgC1A^iii^	4.291(6)	19.7(5)	2.707(4)	3.604(4)	2.329

Symmetry codes: (i) 1 + x, y, z; (ii) -x, 1 − y, −z; (iii) −1 + x, −1 + y, z.

**Table 5:** Number of structures (No.) belonging to each kind of topology found in the CSD database for all the coordination compounds based on the *bpa* ligand, together with a short description and a representative example for each one.

Topology	No.	Description	CSD Refcode
**B**	**234**	**0D**	
B1	205	*Bpa* bridge (*anti*) between 2 metal centers	JUMPUC [51]
B2	6	*Bpa* bridge (*gauche*) between 2 metal centers	UKIBIA [52]
B3	11	Double *bpa* bridge (*gauche*)	WAFTEC [53]
B4	1	Triple *bpa* bridge (*gauche*)	AGACER [54]
B*n*(*X*)	11	*Bpa* bridge (*anti* o *gauche*) between X metal centers	LELMUL [55]
**C**	**450**	**1D**	
C1	251	*Bpa* chain (*anti*)	LADQOW [56]
C2	25	Zig-zag de *bpa* chain (*gauche*)	WUJLUI [57]
C3	25	Double *bpa* bridge chain (*gauche*)	TEJYUC [58]
C4	11	Alternated zig-zag *bpa* chain (gauche and *anti*)	ISIKIE [59]
C5	118	Zig-zag *bpa* chain (*anti*)	DAQFAC [60]
C6	5	B*pa* chain (*anti*) and double *bpa* bridge (*gauche*)	POGLEC [61]
C7	15	Stairs *bpa* (*anti*)	NAMTOK [62]
**L**	**22**	**2D**	
L1	1	Brick *bpa* layer (*anti*)	XOLCOR [63]
L2	6	Herringbone *bpa* layer (*anti*)	NUHLOS [64]
L3	1	Corrugated hexagonal *bpa* layer (*anti*)	TUBBAV [65]
L4	11	Square *bpa* layer (*anti*)	PAKSOK [66]
L5	6	Linear *bpa* layer (*anti*) connected by *bpa* bridge (*anti* or *gauche*)	NAMSEZ [62]
**T**	**12**	**3D**	
T1	6	Cubic *bpa* structure (*anti*)	IDAJUS [67]
T2	3	Diamond like *bpa* structure (*anti*)	HUWDEJ [68]
T3	4	Complex structure of *bpa* layers (*anti*) connected by *bpa* (*gauche*)	VAKDIX [69]

**Table 6:** Parameters of the crystal data, structural resolution and refinement procedure for **1** and **1.ah.**

	1	1.ah
**Formula**	C_22_H_22_Cu_2_N_2_O_12_	C_22_H_12_Cu_2_N_2_O_8_
**FW (gmol^−1^)**	633.49	280.72
**Crystal System.**	Triclinic	Triclinic
**Space Group (n°)**	*P*−1, (2)	*P*−1, (2)
***a* (Å)**	7.2481(11)	5.6282(5)
***b* (Å)**	9.2514(11)	8.6560(5)
***c* (Å)**	9.8569(14)	10.0512(8)
***α* (°)**	115.68(1)	95.739(6)
***β* (°)**	97.80(1)	97.168(7)
***γ* (°)**	96.80(1)	96.154(6)
***V*(Å^3^)**	578.4(1)	479.84(6)
**Z**	1	1
**F (000)**	322	282
***μ* (mm^−1^)**	2.926	3.273
***ρ*_calc_ (gcm^−3^)**	1.819	1.943
**Crystal Size, mm**	0.05 × 0.04 × 0.03	0.12 × 0.07 × 0.06
**Limiting Indices**	−8 ≤ h ≤ 8	−6≤ h ≤ 6
	−10 ≤ k ≤10	−10 ≤ k ≤6
	−11 ≤ l ≤7	−11 ≤ l ≤11
**Reflections Collected**	3163,	3185
**Unique (*R_int_*)**	1831 (0.042)	1693 (0.049)
**Observed [I > 2σ(I)] ^a^**	1560	1527
**Parameters/Restraints**	188/4	154/0
***R*(*F*)/*wR*(*F*^2^) [I > 2σ(I)] ^a^**	0.062/0.159	0.083/0.238
***R*(*F*)/*wR*(*F*^2^) (all data) ^a^**	0.071/0.168	0.088/0.241
**Goodness of Fit on F^2^**	1.064	1.230
**L. Diff. Peak and Hole (e Å^−3^)**	1.194, −1.200	1.656, −1.082

^a^ $\;\;R_{1}=\sum \left|F_{0}\right|\left|\left|F_{0}\right|-\;\left|F_{c}\right|\right|/\sum \left|F_{0}\right|,wR^{2}=\sqrt{\;\sum w{\left(\left|F_{0}\right|-\left|F_{c}\right|\right)}^{2}/\sum w{\left|F_{0}\right|}^{2}}$

## Supplementary Materials

The following are available online at https://www.mdpi.com/1420-3049/24/9/1840/s1, infrared spectra (IR), ORTEP views of asymmetric units of **1** and **1.ah**, XRD profile fitting for **1** heated at 200 °C, comparison between experimental and calculated PXRD patterns for **1** and **1ah**, diffuse reflectance spectra, magnetic susceptibility of **1**.
