# Supplementary material for: Structural Transformations in the Thermal Dehydration of [Cu2(bpa)(btec)(H2O)4]n Coordination Polymer
Source: Molecules. 2019 May 13;24(9):1840. doi: 10.3390/molecules24091840 (PMC6539118; doi:10.3390/molecules24091840)
Supplement: Supplementary file 1 [file molecules-24-01840-s001.pdf]

Supplementary Material

Structural transformations in the thermal dehydration of the $[Cu_2(bpa)(btec)(H_2O)_4]_n$ coordination polymer

Laura Bravo-García, Edurne S. Larrea, Beñat Artetxe, Luis Lezama, Juan M. Gutiérrez-Zorrilla and Maria I. Arriortua

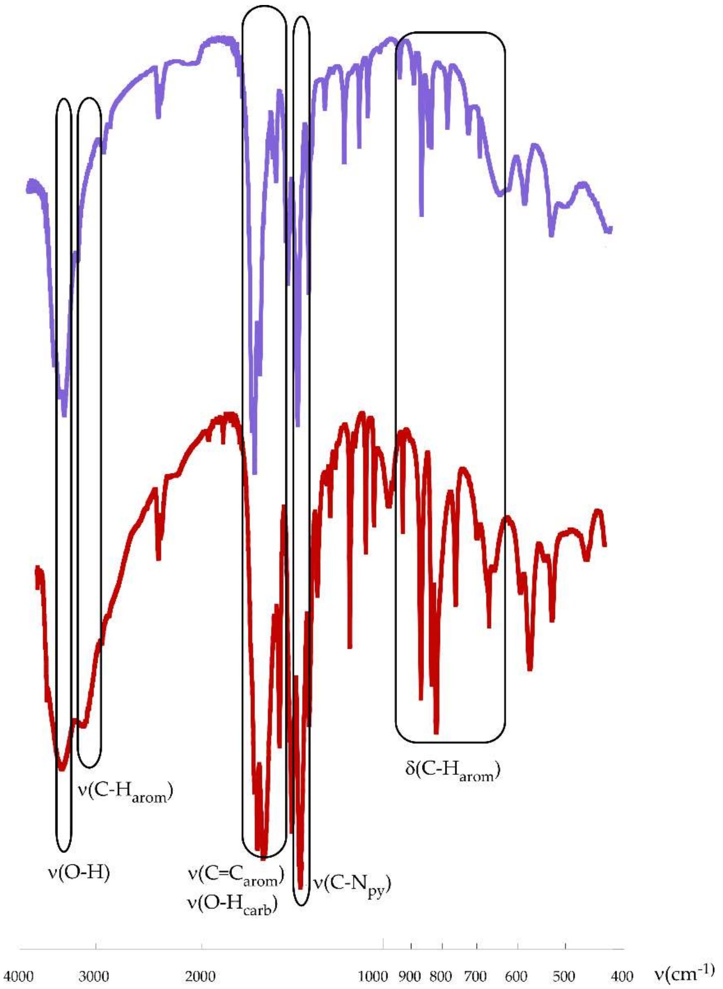

Figure S1. FT-IR spectrum of compound **1** and **1.ah**.

Table S1. Wavenumbers of the most intense bands in the IR spectra of **1** and **1.ah** together with their assignation.

Assignation	Expected wave number	Compound 1	Compound 1ah
ν(O-H)	3650-3200	3430(m)	3371(m)
ν(C-$H_{aromatic}$)	3150-3050	3082(w)	
ν(C=$O_{carboxylic}$)	1700-1600	1620(m)	1625(s)
ν(C=$C_{aromatic}$)	1600, 1580, 1500, 1450	1600(s), 1580(s), 1490(m), 1410(s)	1605(s), 1584(m), 1505 (w), 1462(m)
ν(C=N_{py})	1300	1360(s)	1359(s)
δ(C-$H_{aromatic}$)	950-650	890(m), 800(m), 770(m), 650(m)	857(m), 836(m), 754(m), 672(m)

Intensity: *s* = strong, *m* = medium *w*=weak, Vibrational modes: ν= stretching, δ= bending.

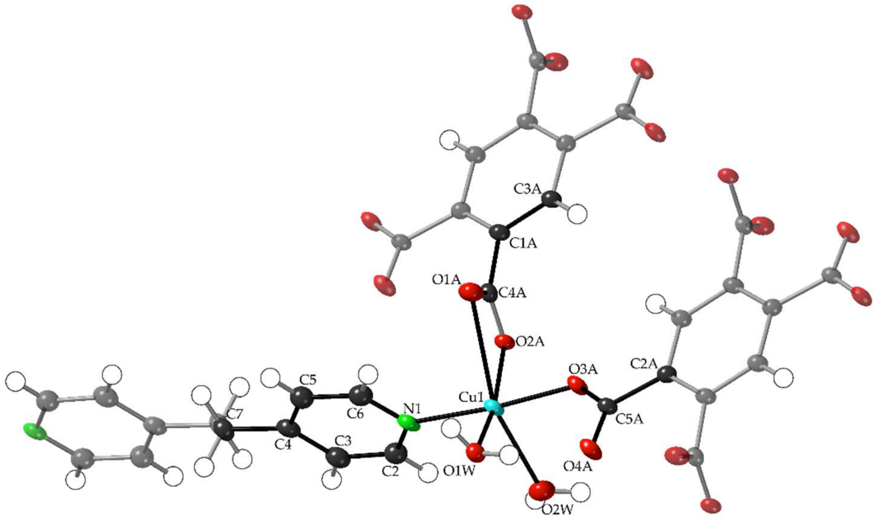

Figure S2. ORTEP[1] view of the coordination sphere of the copper (II) ion in $[Cu_2(bpa)(btec)(H_2O)_4]_n$, (**1**) showing 50% probability ellipsoids, together with atom labelling for the asymmetric unit.

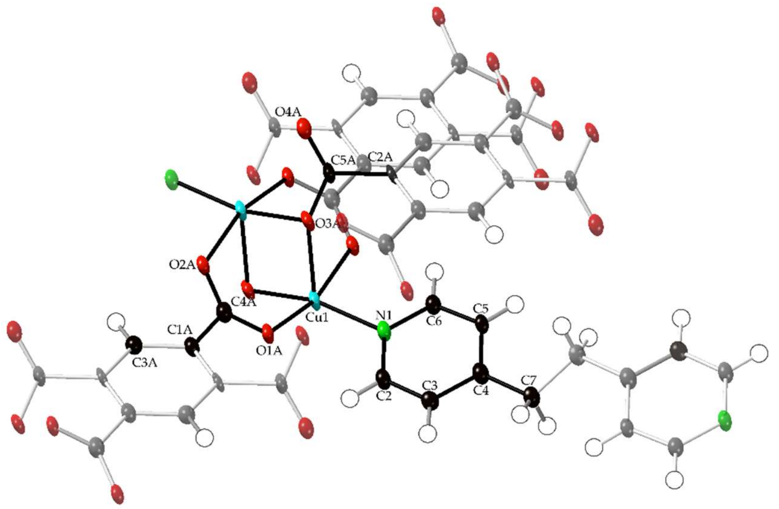

Figure S3. ORTEP[1] view of the copper (II) dimer in $[Cu_2(bpa)(btec)]_n$ (**1.ah**) showing 50% probability ellipsoids, together with atom labelling of the asymmetric unit. Only the ligands belonging to the coordination sphere of one of the copper (II) centers are shown for the sake of clarity.

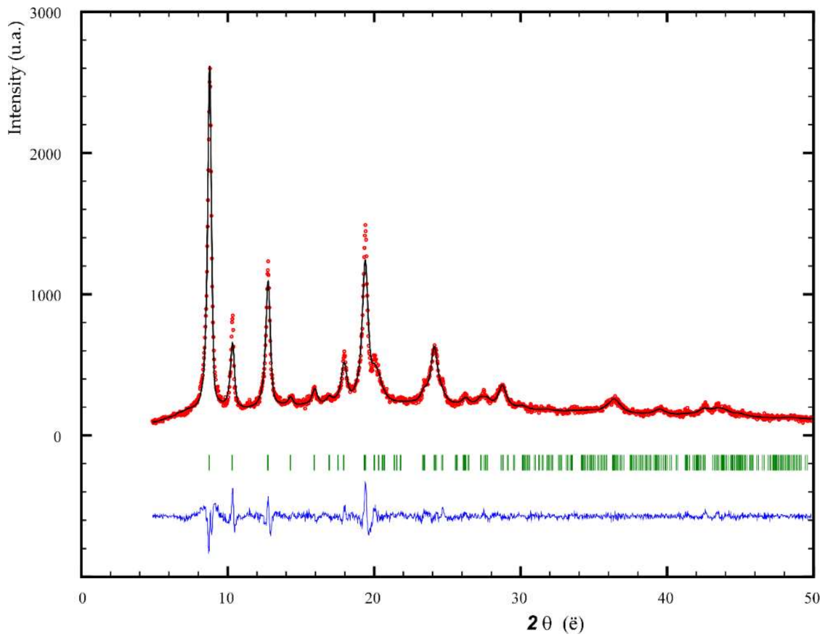

Figure S4. PXRD pattern-matching refinement plot of **1** after being heated at 200°C, using the cell unit parameters of **1.ah**.

Table S2. Cell parameters and agreement factors obtained in the pattern-matching procedure performed on **1** heated at 200 °C.

a **(Å)**	5.6691	***α*** **(°)**	95.601
b **(Å)**	8.6899	***β*** **(°)**	98.381
c **(Å)**	10.2963	***γ*** **(°)**	95.884
V**(Å³)**	495.8978	χ^2	1.62
R_b	6.08	R_p	7.80

$^{a}\ R_{\mathrm{b}} = 100(\sum_{\mathrm{h}}|I_{\mathrm{obs.h}} - I_{\mathrm{cal.h}}|)/(\sum_{\mathrm{h}}|I_{\mathrm{obs.h}}|)$; $R_{\mathrm{p}} = 100(\sum_{\mathrm{i=l,n}}|Y_i - Y_{c,i}|)/(\sum_{i=l,n} y_i)$; $\chi^2 = [R_{wp}/R_{exp}]^2$

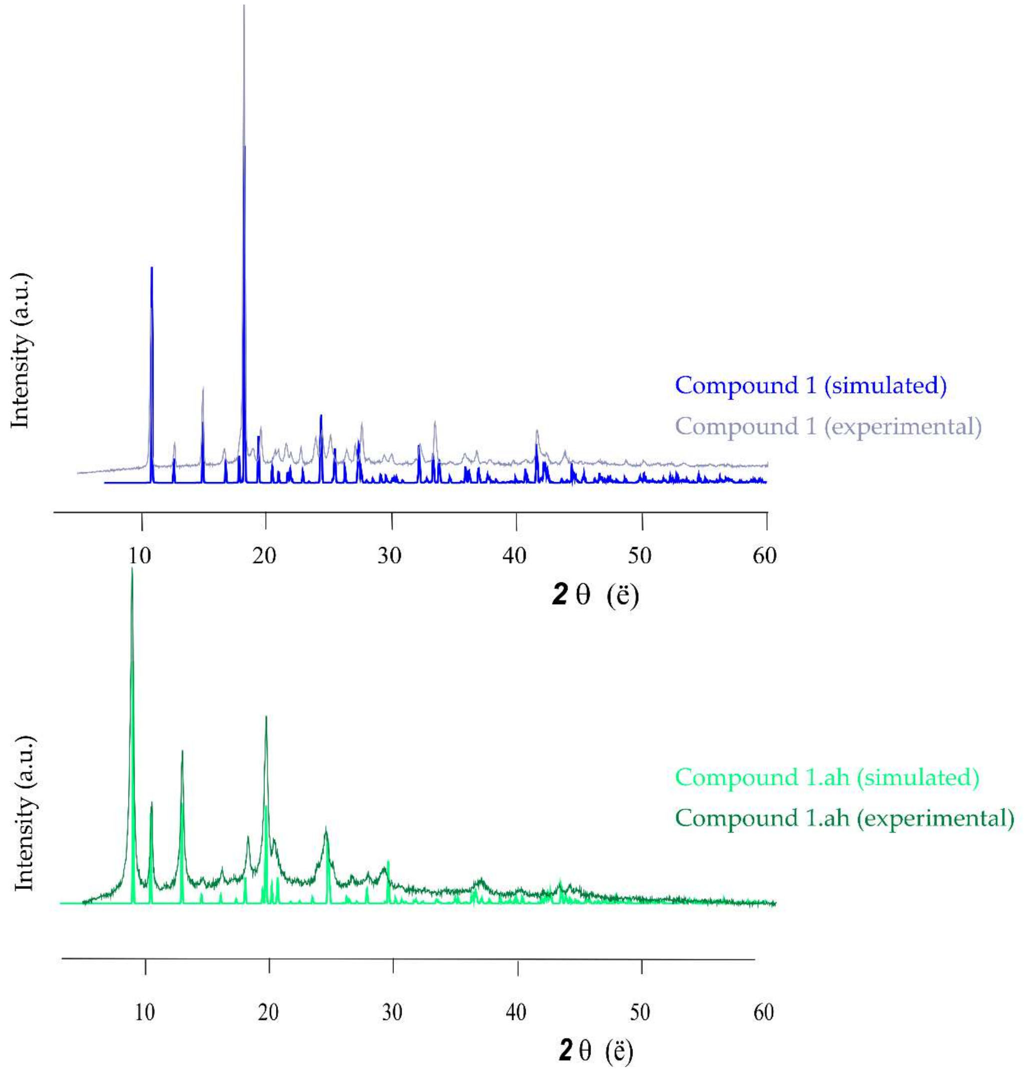

Figure S5. Comparison between experimental and simulated PXRD patterns for compounds **1** and **1.ah**.

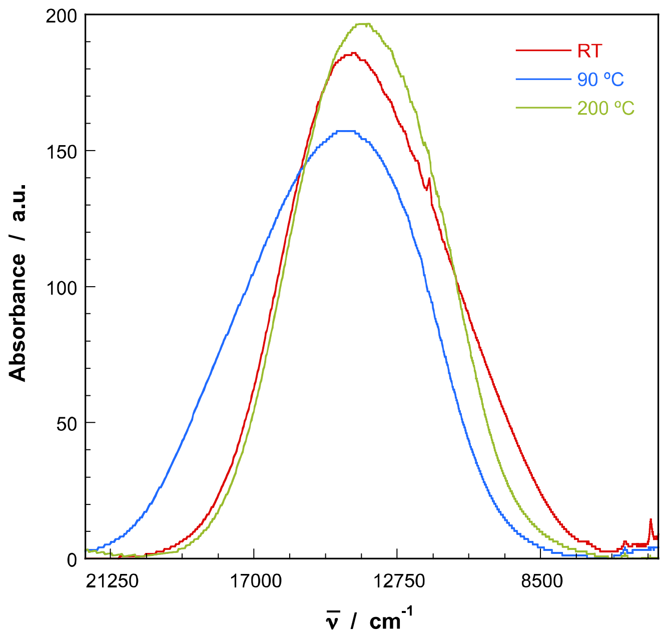

Figure S6. Diffuse reflectance spectra recorded at room temperature on polycristalline samples of **1** and the corresponding dehydrated phases.

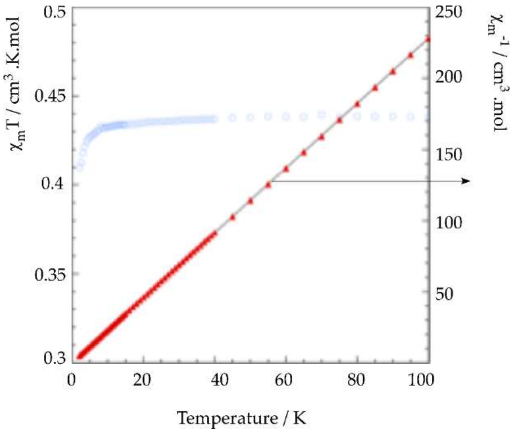

Figure S7. Thermal evolution of $1/\chi_m$ (red triangles) and $\chi_m T$ product (blue circles) for **1**. Fit of the magnetic susceptibility data to the Curie-Weiss law (black solid line)

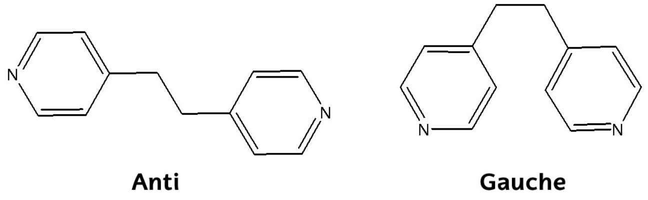

Figure S8. Molecular structures of different extreme conformations (*anti* and *gauche*) for the *bpa* ligand.

REFERENCES: [1] Farrugia, L.J. ORTEP-3 for windows - a version of ORTEP-III with a graphical user interface (GUI). *J. Appl. Crystallogr.* **1997,** *30,* 565.
